# A New Pedestrian Crossing Level of Service (PCLOS) Method for Promoting Safe Pedestrian Crossing in Urban Areas

**DOI:** 10.3390/ijerph18168813

**Published:** 2021-08-20

**Authors:** Tufail Ahmed, Mehdi Moeinaddini, Meshal Almoshaogeh, Arshad Jamal, Imran Nawaz, Fawaz Alharbi

**Affiliations:** 1 UHasselt, Transportation Research Institute (IMOB), Agoralaan, 3590 Diepenbeek, Belgium; tufail.ahmed@uhasselt.be (T.A.); imran.nawaz@uhasselt.be (I.N.); 2 Centre for Public Health, Queen’s University Belfast, Belfast BT7 1NN, UK; M.Moeinaddini@qub.ac.uk; 3 Department of Civil Engineering, College of Engineering, Qassim University, Buraydah 51452, Saudi Arabia; f.a@qec.edu.sa; 4 Department of Civil and Environmental Engineering, College of Design and Built Environment, King Fahd University of Petroleum and Minerals, Dhahran 31261, Saudi Arabia; arshad.jamal@kfupm.edu.sa

**Keywords:** active mobility, walking, urban crosswalk evaluation, crossing indicators, pedestrian safety, public health, level of service analysis

## Abstract

Crosswalks are critical locations in the urban transport network that need to be designed carefully as pedestrians are directly exposed to vehicular traffic. Although various methods are available to evaluate the level of service (LOS) at pedestrian crossings, pedestrian crossing facilities are frequently ignored in assessing crosswalk conditions. This study attempts to provide a comprehensive framework for evaluating crosswalks based on several essential indicators adopted from different guidelines. A new pedestrian crossing level of service (PCLOS) method is introduced in this research, with an aimto promote safe and sustainable operations at such locations. The new PCLOS employs an analytical point system to compare existing street crossing conditions to the guidelines’ standards, taking into account the scores and coefficients of the indicators. The quantitative scores and coefficients of indicators are assigned based on field observations and respondent opinions. The method was tested to evaluate four pedestrian crosswalks in the city of Putrajaya, Malaysia. A total of 17 indicators were selected for the study after a comprehensive literature review. Survey results show that the provision of a zebra crossing was the most critical indicator at the pedestrian crossings, while drainage near crosswalks was regarded as the least important. Four indicators had a coefficient value above 4, indicating that these are very critical pedestrian crossing facilities and significantly impact the calculation of LOS for pedestrian crossings. Four crosswalks were evaluated using the proposed method in Putrajaya, Malaysia. The crosswalk at the Ministry of Domestic Trade Putrajaya got the “PCLOS A”. In contrast, the midblock crossing in front of the Putrajaya Corporation was graded “PCLOS C”. While the remaining two crosswalks were graded as “PCLOS B” crosswalks. Based on the assigned PCLOS grade, the proposed method could also assist in identifying current design and operation issues in existing pedestrian crossings and providing sound policy recommendations for improvements to ensure pedestrian safety.

## 1. Introduction

Walking is a sustainable and healthy mode of transportation that reduces traffic congestion in urban metropolitans. In addition, it has long-term environmental impacts that help conserve energy and achieve a pleasant environment with significantly low air emissions and noise pollution [1]. Additionally, city livability can be increased by improving walking environments and increasing access to public transportation, contributing to the fight against climate change [2]. However, pedestrians represent a significant proportion of vulnerable road users (VRUs) along with motorcyclists and pedal cyclists [3,4,5]. This group of road users is at a potential disadvantage and has no rigid protection barrier against road traffic crash events. According to the WHO report on global road safety, 275,000 pedestrians die globally every year because of traffic collisions [6]. This report also indicated that pedestrians and cyclists represent 26% of the total deaths in road traffic crashes worldwide [6]. Pedestrian casualties account for approximately 14% and over 21% of total road fatalities in the U.S. and the European Union [7,8]. Research has shown that most pedestrians to car collisions happen during road crossing [9,10]. Among pedestrians, the older population is frequently overrepresented among injured pedestrians [11]. Older people are at high risk for several reasons, including their natural declines in perception and visual capacities, relatively weak physical frailty, and reduced mobility, and extended time is usually needed to cross the roadway safely [12,13].

Pedestrians face numerous obstacles during their daily commute, including conflicts with motorized vehicles, turning movements, motorcyclists, other pedestrians, and varying vehicle speeds, all of which compromise their safety, convenience, and comfort [14,15]. They need to be agile as well as cautious in their movement. During the last two decades, urban and transport planners have put more effort to revamp urban areas to make them pedestrian-friendly [16]. Streets are essential in achieving travel sustainability; hence, they should be designed carefully considering the needs of all end-users [17]. Research over the years shows that the quality and quantity of walking can be increased by appropriate design to provide pedestrian-friendly crossing facilities [18]. For example, the construction of new or improved existing crossing facilities can reduce traffic impacts on pedestrians [16].

Different techniques are available to evaluate the pedestrian environment. Some of the most well-known methods include a checklist for assessing the walkability of the route, stated preference technique, and mobile method [18]. A stated preference technique is used to determine pedestrian values to certain specific aspects of walking; a researcher moves on the journey with the person or object that is under research for the direct pedestrian experience in a mobile method for pedestrian studies. Some methods use pedestrian flow, volume, and sidewalk capacity to evaluate streets [16,17,18,19,20]. Evaluation methods are focused on existing conditions and improving the needs of the users, but also help in maintaining a complete street and making it inclusive [21].

The level of service (LOS) method is used for qualitative measurement and evaluation of a service to its user [22]. The LOS method takes different factors into account while measuring the quality of services, such as existing street conditions, pedestrian facilities, and street furniture. Similarly, the pedestrian level of service (PLOS) method evaluates pedestrian facilities on the streets. Some PLOS techniques use a scale measurement from A to F to present the results. “A” shows “free flow” or “very satisfied condition” while “F” reflects “no movement” or “worse” or “very dissatisfied condition”.

Although pedestrian crossing facilities can significantly affect pedestrian safety, there are limited studies evaluating PLOS for pedestrian crossings. Different methods have been developed for midblock crosswalks [23,24]. These methods, however, have serious weaknesses. Some previous studies, for example, evaluated the LOS for crosswalks solely based on pedestrian perception, which can lead to biased results [22]. At the same time, other methods do not consider crossing facilities for pedestrians with disabilities [24]. In the LOS evaluation, the highway capacity manual (HCM)-based approach considers pedestrian volume, motorized vehicle volume, and pedestrian delays while ignoring significant pedestrian crossing facilities.

Based on the literature, most studies have considered the pedestrian level of service for the sidewalks, and there is less attention given to the pedestrian crossing level of service at both the intersection and midblock crosswalks. Additionally, previous studies have only focused on pedestrians’ characteristics such as pedestrian flow, pedestrian area occupancy, pedestrian walking, pedestrian volume, and pedestrian delays; however, they failed to incorporate the pedestrian facilities as an indicator and their standards. Furthermore, these studies have not considered necessary pedestrian crossing facilities, such as speed limits of a vehicle approaching crosswalks, the width of a pedestrian crossing, stop lines for vehicles, crossing orientation, crossing poles, etc., for midblock crosswalks and crossing at an intersection in LOS methods. Besides that, existing methods are inapplicable for evaluating LOS at both intersection crosswalks and midblock crossings simultaneously. Additionally, when developing LOS methods, these methods do not take disabled or elderly pedestrians into account. Therefore, a reliable method is needed for measuring the pedestrian crossing level of service using pedestrian facilities as indicators, which should be flexible enough to meet the diversity of needs of a wide range of pedestrians. This study considers all the vital pedestrian infrastructure facilities for both types of crosswalks by proposing a method to evaluate the LOS for pedestrian crossing. 

The remainder of the paper is structured as follows. Section 2 reviews previous work regarding the pedestrian crossing level of service. Section 3 introduces the research topic. The descriptive statistics of the respondents surveyed are presented in Section 4. The research methods are discussed in Section 5. The findings of the analysis are presented in Section 6. Finally, Section 7 provides discussions on results, summarizes the study’s findings, and gives an outlook for future studies.

## 2. Related Work

Several methods and models are available to measure the pedestrian level of service [17]. Calculating the pedestrian LOS is more complicated compared to calculating the LOS for automobiles [25]. It is essential to review the current research to have a clear picture of the methods available to evaluate the streets.

The very first method in this regard was based on sidewalk capacity and volume [19]. Using design standards as guidelines, the technique was later extended to designing walkways in Bangkok and considered a range of areas and occupancies per person [26]. Similarly, a study conducted by Dowling et al. examined the effect of speed, capacity, and volume to assess PLOS [27]. However, these methods treat pedestrians like vehicles, neglecting some critical factors, such as pedestrian facilities and street furnishings like tactile pavement for disabled users [21]. Researchers have further studied other factors for assessing PLOS, such as the impact of environmental factors [28], comfort, safety and security, attractiveness, continuity, convenience, and system coherence [29].

Seneviratne et al. suggested a level of service theory based on the number of attractions, e.g., malls and restaurants [30]. The problem with these models was that they did not consider the standards and details of the pedestrian facilities available for evaluation. Although the prime purpose of these studies was to suggest valuable indicators for evaluating PLOS, necessary pedestrian infrastructure facilities and amenities such as drinking fountains, tactile pavement, wheelchair accessibility, etc., were not counted in these evaluation methods. It was established that including these pedestrian crossing indicators makes the street more inclusive for walking; however, these are missing in most previous studies [31]. The existing PLOS methods considered only a few pedestrian sidewalk amenities, such as street furniture, trees, street lighting, and drinking fountains [32]. Only a few PLOS techniques consider an acceptable number of sidewalk amenities [17,21,31,33]. In their research, Shekari et al. developed a comprehensive model for assessing PLOS in urban streets concerning pedestrian space [33]. All the critical pedestrian facilities and amenities were taken as an indicator in the model. In another research study, the authors further extended their research and suggested improved PLOS for disabled pedestrians [21]. Another PLOS model by Asadi-Shekari et al. [33] includes main facilities (e.g., curbs, ramps, and sidewalk width), the encouragement of infrastructure facilities (e.g., street lighting, sitting areas for pedestrians, and trees), and convenience facilities (e.g., toilets). Such indicators can transform spaces into more accessible, walkable, and comfortable environments [34].

Like PLOS for sidewalks, researchers have also developed methods for pedestrian crossings. HCM (2000) provides criteria for assessing the pedestrian crossing LOS at both signalized and un-signalized junctions based on pedestrian delays and space requirements. Studies have criticized the method for ignoring crossing facilities (zebra marking, curb extensions, median, etc.) in calculating the LOS at an intersection [21,25]. Even though some studies consider the methodologies proposed by the HCM (2000 and 2010) to be unrepresentative and, more specifically, to overestimate the pedestrian LOS, they are still widely used and widely accepted [35]. Other than pedestrian delay and space needs, several research investigations have identified more contributing variables such as roadway geometric characteristics, traffic flow characteristics, pedestrian volume, area occupancy, walking speed, pedestrian flow, and the bidirectional effect [36,37,38].

Some studies consider the effect of motorized vehicles on pedestrian safety, security, and delays for crosswalks [39,40,41]. Other studies examine the risk of conflict between pedestrians and the left turning of motorized vehicles [42,43]. Goh et al. added pedestrian opinions in developing PCLOS. Factors considered were pedestrian flow, area occupancy, and pedestrian walking speed for bi-directional flows [44]. Their research work was further extended by Bian et al., who defined the standards for proposed indicators [45].

Further studies have explored the effect of motorized vehicle volume, pedestrian delay, vehicle speed, and intersection geometric characteristics on the LOS calculation for the crosswalks [45,46,47]. Pedestrians’ perceptions of crossing a signalized intersection were incorporated to determine the LOS proposed by [46]. Archana et al. also studied the factors that affect PLOS at signalized intersections [14]. The study only considered four crosswalk factors (pedestrian holding area, crosswalk width, crosswalk surface condition, and crosswalk marking). Other crosswalk factors like pedestrian signage, traffic signals at an intersection, poles at crosswalks, etc., were ignored. Furthermore, these methods are limited to crosswalks at an intersection. The factors for midblock pedestrian crosswalks are not incorporated in these studies.

Very few studies have been conducted on the PLOS for midblock crosswalks. Researchers have mainly focused on the PLOS for midblock crosswalks from the perspectives of pedestrian crossing difficulties. Landis et al. designed a method to find the PLOS for midblock street crossings [25]. Even though the method included some pedestrian facilities, such as pedestrian signals, the average speed of a motor vehicle, crosswalk availability, and median, the standards of these facilities were ignored for the LOS calculation; however, these indicators, on the other hand, do not cover a wide range of users (e.g., disabled people) [48]. Similarly, in another study, Chutani et al. also considered the pedestrian difficulty describing the LOS for pedestrian crossing, focusing on the Indian context [49]. In another study, the authors developed a novel LOS method for un-signalized midblock crossings that was based on an opinion survey [50]. Some pedestrian facilities were considered, such as waiting space, crossing distance, crossing markings, median type, and a separate path for a bicycle. A recent study conducted by Kadali et al. proposed a model for the PLOS at unprotected midblock crossings based on pedestrian perceptions [51]. It was found that the perceived PLOS at un-signalized midblock crosswalks can be influenced by land-use conditions, median size, perceived safety, and crossing discomfort, in particular with respect to vehicular traffic-related factors, including the type of vehicles confronted and the number of lanes. However, several other factors, such as comfort and convenience, accessibility, and pedestrian walkway facilities, can influence the PLOS perception. Recent studies indicate the importance of providing zebra crossings, refuge islands, and pedestrian holding spaces at crosswalk locations [48,52,53].

A critical review of the literature suggests that essential indicators in the models [19,40,41] for computing the PCLOS are pedestrian safety, pedestrian–vehicle interaction, and pedestrian perceived crossing difficulty [24,50]. Few studies have explored crossing facility indicators such as speed limits, pedestrian crossing orientation, skid resistance surface, curb ramps, and tactile paving. In addition, the majority of the PCLOS models often overlook items concerning disability issues (e.g., curb ramps, crossing lengths, and tactical paving). Furthermore, previous studies failed to calibrate user perception with infrastructure facilities as an indicator in calculating the LOS for crosswalks to make the method more robust, limiting the use of such techniques to be applicable in all situations. Hence, using these previous methods makes it difficult to determine the essential crossing infrastructure facilities at crosswalks. Thus, an effort is being made to include all vital infrastructure facilities for all types of pedestrians (disabled, elderly) to evaluate crosswalks in the proposed method. The indicators are extracted from various pedestrian crossing guidelines available across different countries, making this method more robust and universally applicable. The existing street condition is compared with standards taken from these guidelines, making it suitable in a different context. The guidelines and the method also help in suggesting improvements for the existing pedestrian conditions.

## 3. Study Area

**Figure 1:**
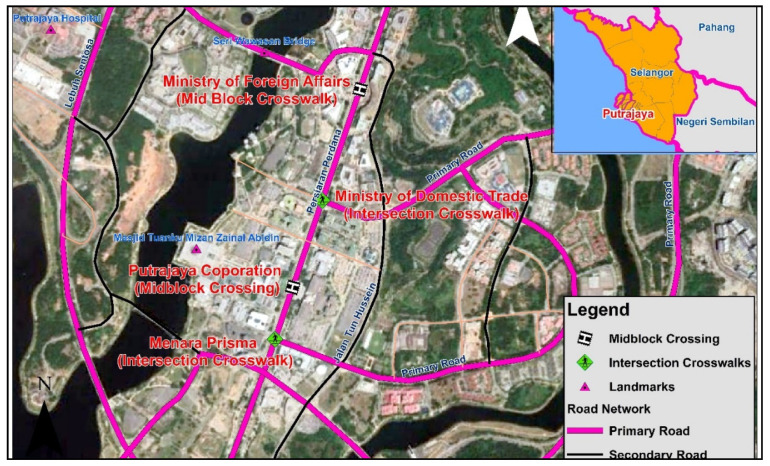
The study area map (Source: Google Earth).

The city of Putrajaya, Malaysia, was selected as the study area. It is a planned city and administrative center of the country located 25 km to the south of the capital city Kuala Lumpur with an area of 49 km^2^ and a population of approximately 0.1 million. Due to overcrowding and congestion, the administrative buildings in the capital were relocated. Putrajaya was built to provide a better urban environment, a good quality of life, and decreased pressure on the capital city’s municipal services. Putrajaya is designed on the neighborhood concept and has proper pedestrian and vehicle movement routes with excellent facilities and connectivity. Four pedestrian crosswalks were selected from Putrajaya Malaysia along urban arterial roads for evaluation based on the proposed method. For this study, the crosswalk at the Ministry of Domestic Trade intersection, Putrajaya Corporation intersection, midblock crossing in front of the Ministry of Home Affairs, and midblock crossing in front of Menara Prisma Putrajaya were selected as case studies for testing the method. The study area can be seen in Figure 1. The chosen crosswalks were simply used to test the proposed LOS. Because Putrajaya is one of Malaysia’s newer cities with good pedestrian infrastructure, the crosswalk situation can range from excellent to average. The crosswalks were chosen to cover the different conditions—the selection of various crosswalk conditions is necessary to test the model’s applicability in a different context. Therefore, we decided to select crosswalks among the excellent ones in terms of providing pedestrian crossing facilities, and the rest having good and average crossing facilities.

## 4. Data Collection

In this study, data were collected from respondents who used pedestrian crosswalks at least once a day to rank pedestrian crossing facilities based on their experience. Table 1 shows the descriptive statistics for the 104 male and 46 female respondents who were surveyed. Male respondents account for 69.33% of all responses, while female respondents account for 30.67%. Table 1 shows that 52.67% of respondents are between the ages of 25 and 30. The number of respondents with graduate degrees were 70, representing 46.67% of the sample group, while survey participants with master’s degrees and doctorate degrees were 66 and 14, representing 44% and 9.33% of the sample population, respectively. Respondents were asked several questions about their daily commute through crossings and their general perception of existing pedestrian crossing facilities. As shown in Table 1, 18%, 13.3%, and 41.33% of individuals in the study sample cross the street twice, three times a day, or more than three times a day, respectively. In comparison, 27.33% of individuals cross the street at least once a day. The majority of respondents stated that they do not feel safe crossing the street. Table 1 shows that a total of 89 respondents agreed with this viewpoint, while the remaining 61 respondents felt safe while crossing the street.

## 5. Materials and Methods

For data collection in pedestrian crossing studies, some conventional techniques used by researchers are direct observation [16,20,37], video techniques [21,27], and questionnaires [12,42,43]. Using only direct observation can generate biased results because it is purely dependent on the researcher’s perception. Similarly, using only a questionnaire method of data collection limits the results to the respondent’s perception. Combining both these methods would undoubtedly remove biases and perceptions in the evaluation process. This study uses an audit tool where the indicators are directly observed at the study area as well as a questionnaire method for knowing the importance of the selected indicators.

Historically, three methods of data analysis are prevalent among researchers, namely, regression analysis [25,46,54], simulation [55], and point systems [29,56,57,58,59]. Simulation studies usually have calibration problems. If there are unacceptable differences between the model results and the validation data, then the model has to be reconstructed again, and sometimes it requires modification of the indicators for the reconstruction of the model [60]. The point system developed by Dixson et al. for the PLOS is helpful for rating street conditions [58]. However, the weights of the various indicators in this method are chosen arbitrarily.

Additionally, there are no separate score categories for multiple situations. Gallin et al. also used a rating system to evaluate the PLOS [59]. Again, the strength and weight of an indicator are based on personal judgment, which increases the possibility of biased results. On the other hand, the point system method can be improved by including more indicators and considering different street conditions. The point system is modified to cover the objectives of this PCLOS study, which are focused on pedestrian crossing facilities. In addition, the point-scoring method can adjust different situations in the process. Therefore, it can easily be connected with the design process of the streets.

### 5.1. Indicators Selection

This study aims to consider most pedestrian crossing facilities for people with different needs and abilities. The identification is made through analyzing the comprehensive literature of existing pedestrian street crossing guidelines from various cities and crosswalk assessment tools (audits and questionnaires). This is needed to determine the essential elements for inclusion in a first effort to build an assessment tool for analyzing pedestrian crossings. The review of various guidelines and audit tools for pedestrian crossing is necessary for recognizing important crossing indicators. In addition, the reason for selecting indicators from various countries and cities is instrumental in covering different contexts. Moreover, this method can be applied to any crosswalk. After a thorough literature review of current urban pedestrian street crossing guidelines implemented in different countries, 17 indicators were selected for this study. All vital pedestrian crossing facilities are considered, which can significantly improve pedestrian safety when crossing urban roads and accommodating pedestrians with diverse abilities, i.e., the disabled. The process of reviewing literature and selecting indicators was continued until the indicators were repeated. Table 2 shows the indicators chosen for this study and the references for these chosen indicators.

### 5.2. Method

This study proposes a PCLOS technique based on a point system to rate pedestrian crossings facilities. Most of the previous studies that followed the point system to estimate the level of service for pedestrians used observation as the primary tool to calculate the scores for the related indicators [17,21,58,59,61]. Some of the new efforts in this area also include the population and pedestrian-related ideas by considering some questionnaires and using the results as a coefficient for the observed scores [32,34,48,62]. The current study also follows the same approach and adds a questionnaire to estimate the importance of the indicators, while keeping observation as the primary tool to calculate the scores. After finalizing the indicators for the study, an audit tool is created by integrating all of the valuable indicators gathered from various sources. The determination of standards is crucial to score the pedestrian crossing facilities. The standard of every indicator is determined in the audit tool. The scoring framework used in this research is designed to minimize subjectivity. Indicators are assigned a score through the point score method. The scores of indicators are based on the standards gathered at the end of the second stage. Table 3 shows the audit tool for this study where pedestrian crossing indicators and their score based on standards and guidelines are listed. Based on the standards, the indicators are assigned a score of 0, 0.5, or 1 (see Table 3). All observers who desire to use this structure will find it simple to determine the appropriate score for each indicator. In addition, using this structure, the likelihood of different observers considering different scores for the same situation is very low.

These indicators were then used to develop a questionnaire for data collection, emphasizing the significance of these indicators for pedestrian crossings. The importance of each indicator was estimated by using a 5-point Likert scale in this study. Respondents could rate pedestrian crossing indicators from 1 to 5 based on their importance. Scale 1 represents the least important indicator, while scale 5 shows the extremely important indicators. The questionnaire results were used to calculate the weight for each crosswalk indicator.

Finally, field measurements were taken in the four pedestrian crossings on arterial roads in Putrajaya, Malaysia to apply the methodology and calculate the PCLOS based on the proposed method.

### 5.3. Mathematical Definition

Because each of the 17 indicators uniquely affects the PCLOS, they may have specific coefficients that resulted in developing the following PCLOS model shown in Equation (1). The model is inspired by the previously developed PLOS model, bicycle safety index, neighborhood sidewalks assessment tool, and comfort walkability index [17,32,34,63]. The model included all of the essential indicators identified from the literature for pedestrian crossing.

(1) $$\mathrm{PCLOS}=\overset{n}{\underset{i=1}{\sum }}C_{i}P_{i}$$

where PCLOS is the pedestrian crossing level of service, *i* is the number of each indicator, *C_i_* is the coefficient of pedestrian crossing indicators, *P_i_* is the pedestrian indicator score, and *n* is the total number of indicators.

*C_i_* represents the coefficient of the indicators and it is different for each indicator. The coefficient of indicator (*C_i_*) shows the importance and priority of the indicators in the calculation of the PCLOS. This coefficient was estimated using a questionnaire survey from field experts and pedestrians. The mean of each indicator represents the coefficient of the indicator. The sample size of this study was 150, with a 95% confidence level and a sampling error of 8%. The sample size was calculated using the Krejcie and Morgan method. In total, 20 field experts and 130 pedestrian crosswalks users were consulted/surveyed for the current study in the questionnaire survey. Field experts were interviewed because it was essential to know their opinion on the critical pedestrian crossing facilities. A sampling error of 8% is acceptable because the ratings to indicators were based on their experience rather than just perception. The questionnaire was uploaded and disseminated online through social media sites so that anybody interested in the survey could take part in it. The reason for sharing the questionnaire through social media sites is to get a wide range of responses from the respondents with different social and educational backgrounds who use pedestrian crossing facilities. It was essential to also know the expert’s point of view regarding the crossing facilities, as this can effectively increase pedestrian crossing safety. Transport planners, urban planners, and transportation engineers were regarded as experts in this study.

Similarly, *P_i_* illustrates the pedestrian indicator score. It is calculated by comparing existing pedestrian crossing facilities to the standards outlined in the guidelines developed for each indicator. Based on the standards, the indicators are assigned a score of 0, 0.5, or 1. For example, an indicator that was available in pedestrian crossing and according to standard was given a score of 1. In contrast score of 0 was assigned to the indicators that are not available in the crosswalks. Furthermore, the indicators found in crosswalks that did not conform to the standard were given a 0.5 score. Table 3 illustrates pedestrian crossing indicators and their score based on the standards.

After calculating the PCLOS based on Equation (1), the PCLOS% can be defined for the pedestrian crosswalk. The PCLOS% is used for interpreting the results of the pedestrian crossing. It compares the existing pedestrian crossing indicators with the ideal condition. The percentage of the PCLOS was calculated using the expression below (Equation (2))

(2) $$\mathrm{PCLOS}=\overset{n}{\underset{i=1}{\sum }}C_{i}P_{i}$$

where the PCLOS% is the pedestrian crossing level of service percentage. All other variables are the same as explained above.

Table 4 shows the categories of the PCLOS% rating and its interpretation. For example, the PCLOS% rating “A” represents the best or almost ideal pedestrian crosswalks where most pedestrian crossing facilities are present. In contrast, the PCLOS% rating “F” represents the worst-case scenario for the pedestrian crossing where no pedestrian crossing facility is available.

The method adopted in this study has significant advantages. Previous PCLOS studies have often overlooked indicators for people with a disability and impairment issues, such as space between bollards, curb ramps, crossing lengths, and tactical paving [14,18,29]. Besides that, most current PCLOS methodologies cover a limited set of indicators and pedestrian specifications. As a result, the existing methods do not have a universal application and may not be appropriate in all circumstances [32]. However, the current study considers most pedestrian crossing facilities for people with different needs and abilities. In the past, the PCLOS model has covered a narrow range of indicators and pedestrian requirements. Current methods do not incorporate a sufficient number of crosswalk facilities; thus, the suggested technique tackles this deficiency by incorporating the broadest range of crosswalk design factors. In addition, this study specifically looked at microscale design factors at the crosswalk level, which has never been done before. Consequently, this research used a simplified mathematical point system that produced the desired results to assess the crosswalks. The point system is also considered the intermediate condition for the crosswalk indicators.

## 6. Results

**Figure 2:**
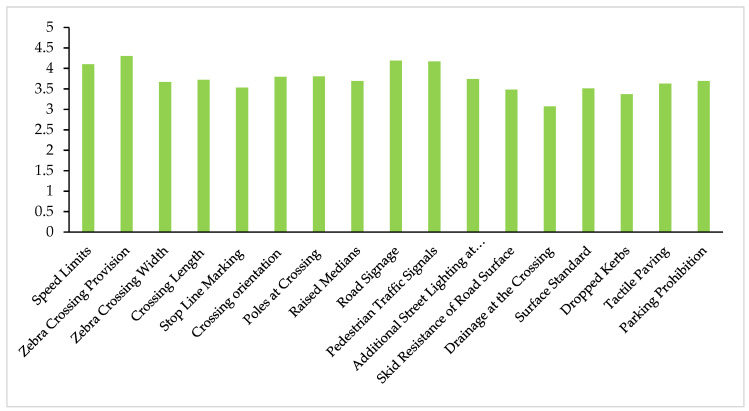
Coefficient of indicators estimated using a questionnaire survey.

The coefficient of the indicators was estimated from the data collected from respondents. These coefficients represent the importance of the indicators. Different indicators have different effects on the PCLOS, so it was essential to find the significance of each indicator. The respondents rated all the indicators from 1 to 5. The mean of each indicator shows its coefficient. The higher the coefficient is, the more critical the indicators are in a pedestrian crossing. Figure 2 represents the coefficient, maximum value, and minimum values of the indicators. The value of the coefficient was further used in the calculation of the PCLOS. Based on Table 3, the most critical crossing facility indicator was providing a zebra crossing, while the least critical facility to the pedestrian was drainage at the crossing. Figure 2 shows the Ci for each indicator calculated from the survey results.

All coefficients of the pedestrian crossing indicators and Pi were calculated based on Table 1 and Table 2. As a result, the PLCOS, PCLOS%, and PLOS grades for the Ministry of Domestic Trade intersection were found (using Equations (1) and (2) and Table 3). Typical results for calculating the crossing LOS are shown below (Equation (3)).

(3) $$\begin{array}{l} \mathrm{PCLOS}=\left(4.10\times 1\right)+\left(4.30\times 1\;\;\right)+\left(\;3.67\times 1\;\right)+\left(3.72\times 0.5\;\right)\\ +\left(3.53\times 0.5\right)+\left(3.79\times 1\right)+\left(\;3.80\times 0.5\right)+\left(\;3.69\times 1\right)\\ +\left(4.19\times 1\right)+\left(4.17\times 1\right)+\left(\;3.74\times 1\right)+\left(3.48\times 0.5\right)\\ +\left(3.07\times 1\right)+\left(\;3.51\times 1\right)+\left(3.37\times 0.5\right)+\left(3.63\times 0\right)\\ +\left(3.69\times 1\right) \end{array}$$

The above calculations yield a PCLOS = 52.63. The PCLOS% can be then computed as: PCLOS% = (52.63/63.45) × 100 = 82.95. Referring to Table 4, the PLOS grade for a pedestrian crossing at the Ministry of Domestic Trade Intersection is A. The PLCOS, PCLOS%, and PCLOS grades for midblock crossing in front of the Ministry of Home Affairs are defined based on Equations (1) and (2) and Table 4, respectively.

Based on the Ministry of Domestic Trade intersection crosswalk results in Putrajaya, Malaysia, the suggested improvements include reducing the crossing length to four lanes. It can be done by extending the curbs before the location of the crosswalk with the parking allowed. The pedestrian crossing poles were not according to the standards. The average distance between the poles at the crosswalk was 0.13 m, which is insufficient for accommodating pedestrians who use wheelchairs. Hence, it is recommended that the distance between pedestrian crossing poles should be increased to 1.2 m.

Additionally, the skid resistance surface in front of the crosswalk should be increased from 25 m to 50 m. The dropped curbs/curb ramp width should be increased to at least 1.2 m. Tactile paving was not available at the crosswalk location, but it is recommended that it be installed with a minimum width of 0.30 m and a distance of 0.60–0.80 m from the edge of the footpath.

Based on the pedestrian indicators score from field observation, and coefficient of indicators (refer to Figure 1), the PCLOS at the midblock pedestrian crossing in front of Ministry of Home Affairs Putrajaya is calculated as follows (Equation (4)).

(4) $$\begin{array}{l} \mathrm{PCLOS}=\left(4.10\times 1\right)+\left(4.30\times 1\;\;\right)+\left(\;3.67\times 1\;\right)+\left(3.72\times 0\;.5\right)\\ +\left(3.53\times 1\right)+\left(3.79\times 1\right)+\left(\;3.80\times 0.5\right)+\left(3.69\times 1\right)\\ +\left(4.19\times 1\right)+\left(4.17\times 1\right)+\left(\;3.74\times 0.5\right)+\left(3.48\times 0\right)\\ +\left(3.07\times 1\right)+\left(\;3.51\times 1\right)+\left(3.37\times 0.5\right)+\left(3.63\times 0\right)\\ +\left(3.69\times 1\right) \end{array}$$

The above calculations lead to a score of PCLOS = 49.02. The PCLOS% may be then similarly calculated from the relation: PCLOS% = (49.02/63.45) × 100 = 77.26, which refers to a score of grade B for this crossing (see Table 4).

Improvements suggested at the midblock crossing in front of the Ministry of Home Affairs include to reduce the number of lanes to four at the crosswalk location, reducing the crossing length for the pedestrians. Recommendations for poles at the crossing, dropped curbs, and tactile paving is the same as the recommendations for the midblock crossing at the Ministry of Home Affairs, Putrajaya. In addition, streetlights were available with a 27 m gap between two streetlights, which is not according to standards. This warrants the installation of additional streetlights at the crosswalk locations. It is also recommended to provide a minimum skid resistance length of 50 m upstream of the pedestrian crossing. Table 5 shows the PCLOS score, PCLOS percentage, and assigned PCLOS grade to the pedestrian crosswalks selected for the study.

The PCLOS calculation for the intersection crossing at Menara Prisma Putrajaya is as follows (refer to Equation (5)).

(5) $$\begin{array}{l} \mathrm{PCLOS}=\left(4.10\times 1\right)+\left(4.3\times 1\;\;\right)+\left(\;3.67\times 1\;\right)+\left(3.72\times 0.5\right)\\ +\left(3.53\times 0.5\right)+\left(3.79\times 1\right)+\left(\;3.80\times 0\right)+\left(3.69\times 1\right)\\ +\left(4.19\times 0\right)+\left(4.17\times 1\right)+\left(\;3.74\times 0\right)+\left(3.48\times 0\right)\\ +\left(3.07\times 1\right)+\left(\;3.51\times 0.5\right)+\left(3.37\times 1\right)+\left(3.63\times 0\right)\\ +\left(3.69\times 1\right) \end{array}$$

The above calculations yield PCLOS = 39.26. Based on Equation (2), the PCLOS % can be then computed as PCLOS % = (39.23/63.45) × 100 = 61.82. Referring to Table 4, the PLOS grade for the Menara Prisma Putrajaya intersection crossing is B.

Improvements suggested for the Menara Prisma Putrajaya intersection based on data after the results include that the road width should be reduced. There are currently six lanes and it is recommended to reduce the number of lanes to four at the crosswalk location. There are no poles and bollards at the crosswalk’s location; hence, poles are recommended with a height between 0.75 and 1.2 m, as well as being 75 mm wide and having a minimum gap between poles of 1.2 m to accommodate disabled pedestrians using wheelchairs. Furthermore, a better skid resistance surface for the vehicle should be provided before approaching the crosswalk. In addition, the provision of tactile paving would further improve the safety of disabled pedestrians at the crosswalk. Along with that, traffic signs are encouraged to be installed for informing drivers about the approaching crosswalk.

The PCLOS at the midblock pedestrian crossing in front of the Putrajaya Corporation is calculated based on Equation (1) and the pedestrian indicator scores from the field observation and the coefficient of indicators (refer to Figure 1).

(6) $$\begin{array}{l} \mathrm{PCLOS}=\left(4.10\times 1\right)+\left(4.3\times 1\;\;\right)+\left(\;3.67\times 1\;\right)+\left(3.72\times 0.5\right)\\ +\left(3.53\times 1\right)+\left(3.79\times 1\right)+\left(\;3.80\times 0.5\right)+\left(3.69\times 0.5\right)\\ +\left(4.19\times 0.5\right)+\left(4.17\times 0\right)+\left(\;3.74\times 0\right)+\left(3.48\times 0\right)\\ +\left(3.07\times 0.5\right)+\left(\;3.51\times 0.5\right)+\left(3.37\times 1\right)+\left(3.63\times 0\right)\\ +\left(3.69\times 1\right) \end{array}$$

Based on the above calculations, the PCLOS = 37.44. The computed PCLOS% (37.44/63.45) × 100 = 59.00. So, the PLOS grade for this intersection is C (refer to Table 4)

Improvements suggested for midblock crossing in front of Putrajaya Corporation are providing a pedestrian traffic signal, installing additional streetlights for better visibility at night, and providing a skid resistance surface before the crosswalk. In addition, the average distance between the poles at the crosswalk was 0.12 m, which is very narrow and cannot accommodate pedestrians with wheelchairs. Hence, the distance between the poles should be increased by 1.2 m. Only no parking traffic signs are provided before the crosswalk, and it is suggested to install a crosswalk sign before the crosswalk. In addition, the road length should be reduced to four lanes at the crosswalk to reduce the crosswalk length for pedestrians, which will increase pedestrians’ safety. Moreover, tactile paving, drainage at the crossing, and surface standards are also recommended for improvement. 

Table 6 shows a summary of both the case studies based on Equations (1) and (2) and Table 3. Two types of analyses were done to understand the results based on the data collected from pedestrian crossing case studies and respondents. First, descriptive statistical analysis was used to estimate the population characteristics of the respondents. Furthermore, the coefficient of the pedestrian indicators was also estimated. After that, scores were assigned to every case study indicator based on the data collected through direct observation. Then, the scores were assigned after comparing the collected pedestrian crossing facilities’ data and the standards. Finally, the coefficient and the scores of indicators were used to calculate the PCLOS, PCLOS%, and PCLOS grades for each case study.

## 7. Conclusions, Discussion, and Outlook

Walking is one of the active transport modes with numerous benefits for long-term sustainable transportation and public health improvement. However, pedestrians represent a significant proportion of VRUs worldwide. Crosswalks/pedestrian crossings are essential locations on the road. It is critical to provide appropriate pedestrian crossing facilities to make crosswalks safer for pedestrians. Crosswalk evaluation methods are vital for improving the quality of urban streets and indicate the potential for improvements. Previous studies in this regard have considered a limited range of pedestrian facilities and abilities [14,49,50] that make them insufficient to maintain more inclusive and pedestrian-friendly streets; transportation planners should consider diverse urban populations and crosswalks facilities.

Moreover, various studies have highlighted that the currently available methods of analysis do not include the right combination of associated factors for the facility evaluation process in pedestrian LOS studies [48]. In addition, some of the models do not consider users’ perceptions and only focus on the guidelines and literature [17,21]. This study proposes a holistic PCLOS framework for evaluating crosswalks based on a wide range of universal guidelines and standards from the existing literature that meet the needs of pedestrians by considering various facilities and incorporating pedestrian perceptions in the model.

The practical PCLOS method introduced in this study was used to assess intersection and midblock crossings for four case studies in Putrajaya in Malaysia. The proposed PCLOS method is based on the presence of the pedestrian crossing indicators and their coefficients. A total of 17 indicators were selected for the study after a comprehensive literature review of pedestrian crossing guidelines. Indicators’ quantitative scores were assigned based on the field observation of the selected sites. The coefficients of the indicators were calculated based on the questionnaire survey. Four indicators had a coefficient value above 4, indicating that these are essential pedestrian crossing facilities and have a very high impact on the LOS calculation for pedestrian crossings. These indicators are speed limits, zebra crossing provisions, road signage, and pedestrian traffic signals.

Furthermore, all the 17 indicators have a coefficient value above three, indicating that all these facilities are essential at crosswalk locations. Questionnaire survey results showed that the provision of a zebra crossing was perceived as the most critical crossing facility, while having a drainage facility was regarded as the least important. The proposed PCLOS method was used to establish qualitative service ratings (A to F) for the considered crosswalk facilities to help urban planners prioritize facilities for potential improvement. The proposed PCLOS framework is applicable in other cities for the adequate maintenance and safe and sustainable growth of urban streets since it is based on universal standards from developed guidelines. For the current study, the questionnaire was uploaded and disseminated online through social media sites. Therefore, the majority of our respondents were young people. The questionnaire survey results can affect the coefficients (importance of the indicators) of the indicators. It is recommended for further studies to explore the effects of including the older population on the coefficients. In addition, the emphasis of this work is on design-related variables to develop some universal assessment tool, and the scores are estimated through observation. Including weather and time of day in the methodology could be an exciting suggestion for future research to propose a dynamic LOS.

The proposed PCLOS method can further be developed for bicycle users as well. Crossing facilities for bicycles were not considered in this study. It can be developed by adopting similar analysis methods used in this study. Similarly, the current LOS method is based on assessing the crosswalk at the urban arterial road. The standards are different for the type of urban roads; for example, the speed limits may vary for the different typologies of roads. The standards are considered only for arterial roads. A similar street-crossing methodology can be developed for other types of streets as well. In addition, future studies that are interested in the perceptions and differences of indicators for pedestrians should consider increasing the sample size.

Furthermore, this model for evaluating pedestrian crossing can further be improved by adding more indicators. Most of the critical pedestrian crossing facilities are incorporated in the study. However, the methodology is flexible in adding more indicators, which can further improve the model. In the future, current methods could consider other crossing facilities and pedestrian attributes that will yield more generic results and be more practical for different geographic locations.

## Figures and Tables

**Table 1:** Descriptive statistics of the data.

Sr. No	Demographic	Categories	Frequency	Percentage (%)
1	Gender	Female	46	30.67
		Male	104	69.33
2	Age	19–24	40	26.67
		25–30	79	52.67
		31–36	17	11.33
		Above 36	14	9.33
3	Education	Doctorate	14	9.33
		Graduation	70	46.67
		Master	66	44.00
	Pedestrian Safety			
4	Pedestrian Crossing Frequency	More than three times a day	62	41.33
		Thrice a day	20	13.33
		Twice a day	27	18.00
		One time a day	41	27.33
5	Safety During Crossing	No	89	59.33
		Yes	61	40.67

**Table 2:** Selected PCLOS indicators.

Sr. No	Indicators	Guidelines
**1**	Speed Limits	Pedestrian Crossing Specification and Guidance, National Roads Authority Ireland
**2**	Zebra Crossing Provision	Crossing facilities for pedestrians, New Zealand Transport Agency
**3**	Crosswalk Width	The Design of Pedestrian Crossings Department of Transport UK
**4**	Crossing Length	Guide Information for Pedestrian Facilities Austroads
**5**	Stop Line Marking	Guide Information for Pedestrian Facilities Austroads
**6**	Crossing Orientation	Guide Information for Pedestrian Facilities Austroads
**7**	Poles/Bollards at Crossing	Crossing facilities for pedestrians New Zealand Transport Agency
**8**	Refuge Island/Raised Medians	Planning and designing for pedestrians: guidelines from the Department of Transport Western Australia
**9**	Road Signage	Planning and designing for pedestrians: guidelines from the Department of Transport Western Australia
**10**	Pedestrian Traffic Signals	Subdivision and development street standards Pima County, US
**11**	Additional Street Lighting at Crossing Points	Pedestrian Crossing Specification and Guidance, National Roads Authority Ireland
**12**	Skid Resistance of Road Surface	Pedestrian Crossing Specification and Guidance, National Roads Authority Ireland
**13**	Drainage at the Crossing	Pedestrian Crossing Specification and Guidance, National Roads Authority Ireland
**14**	Surface Standard	The Design of Pedestrian Crossings Department of Transport UK
**15**	Dropped Curbs/Curb Ramp	Crossing facilities for pedestrians, New Zealand Transport Agency
**16**	Tactile Paving	Mobility Master Plan Bicycle and Pedestrian Design Guidelines, Tacoma Washington
**17**	Parking Prohibition when Curb Extension is not Provided	Crossing facilities for pedestrians, New Zealand Transport Agency

**Table 3:** Pedestrian crossing indicators and their scores based on standard guidelines.

Sr. No	Facility	Score	Standards
**1**	Speed Limits	P1 = 1 if speed ≤ 45 mph P1 = 0 if speed > 45 mph	On arterial streets, the speed limits should be 45 mph (72 kph)
**2**	Zebra Crossing Provision	P2 = 1 if available and according to standards P2 = 0.5 available but not according to standard P2 = 0 if zebra crossing is not available	Zebra crossings should not usually be sited (1) Within 100 m of (a)any other pedestrian crossing point on the same route (b) At major intersection unless located at the intersection(c) At a signalized pedestrian crossing (2) Near speed humps, unless they are combined with the speed hump (as a platform) (3) Spacing between the two strips of marking should be 0.3–1.5 m; (4) Recommended strip width is 0.3–0.6
**3**	Crosswalk Width	P3 = 1 if width >2.4 m P3 = 0.5 if width = 2.4 m P3 = 0 if width < 2.4 m	Minimum desirable crosswalk width for a pedestrian flow less than 600 pedestrians per h If the flow is more than 600 per h, then the width of 5 m is recommended
**4**	Crossing Length	P4 = 1 if no. of lanes ≤ 4 P4 = 0.5 if no. of lanes 5–6 P4 = 0 if no. of lanes <6	It can be reduced by extending the footpath and/or by providing pedestrian refuges. A pedestrian refuge is desirable on all roads with moderate to high traffic volumes (e.g., two-lane two-way roads), high pedestrian crossing volume, or a high proportion of people with disabilities
**5**	Stop Line Marking	P5 = 1 if according to the standards P5 = 0.5 available but not according to standards P5 = 0 if not available	(1) Should be provided in advance of crossings on the road that have at least two travel lanes in each direction (2) Stop lines should be used from 1 to 15 m in advance of the pedestrian crosswalk (3) Recommended strip width is 0.3–0.6 m
**6**	Crossing Orientation	P6 = 1 if the crossing is at a right angle to the road P6 = 0 if the crossing is not a right angle to the road	Crosswalk markings should be at 90 degrees to the street to designate the shortest path for crossing and minimize pedestrian exposure.
**7**	Poles/Bollards at Crossing	P7 = 0.5 if available but not according to standards P7 = 1 if according to the standards P7 = 0 if not available	(1) Black and white (preferably reflectorized) striped poles, (2) The height should be between 0.75 and 1.2 m and 75 mm wide (3) Minimum gap between poles should be 1.2 m for the wheelchair users (4) The space between the curb and poles should 0.45 m from the curb
**8**	Refuge Island/Raised Medians	P3 = 1 if available and according to standards P3 = 0.5 if available and according to standards P3 = 0 if not available	(1) Medians and refuge islands should be a desirable width of 2.4 to 3 m (8 to 10 feet) wide and a minimum width of 1.8 m (6 feet) (2) Absolute minimum depth of 1.5 m minimum and absolute minimum width of 1.2 m (3) Medians or refuge islands are recommended whenever crossing distances exceed 18.3 m (60 feet)
**9**	Road Signage	P9 = if available according to standards P9 = if available but not according to standards P9 = if not available	Any of the following two should be available (1) Advance pedestrian crossing signs: these signs should not be mounted with another warning sign (except for a supplemental distance sign or an advisory speed plate) or regulatory sign (except for NO PARKING signs) (2) Pedestrian crossing sign: this sign should be used only at the crosswalk location and not in advance of it. (3) Both signs should be equipped with internal lighting for increased visibility at night.
**10**	Pedestrian Traffic Signals	P10 = 1 if available according to standards P10 = 0.5 if available but not according to standards P10 = 0 if not available	(1) Needed for major arterials road (2) Needed for midblock crossing if a road has four or more lanes (3) It is preferable to place device not closer than 0.75 m and a maximum of 3 m from the curb (4) It should not more than 1.5 m from crossing
**11**	Additional Street Lighting at Crossing Points	P11 = 1 if enough streetlights are available P11 = 0.5 if not enough streetlights are available P11 = 0 if no streetlights available	(1) Enough streetlights should be provided (2) The streetlight poles should be max 9 m apart from each other (3) Additional streetlights should be provided to increase night visibility
**12**	Skid Resistance of Road Surface	P12 = 1 if available according to standards P12 = 0.5 if available but not according to standards P12 = 0 if not available	The minimum length of skid resistance should be 50 m on approaching zebra and signal-controlled crossings with a speed of 50 km/h.
**13**	Drainage at the Crossing	P13 = 1 if there is drainage at the crossing P13 = 0 if there is no drainage at the crossing	Drainage problems lead to ponding at the crossing points, which could be a particular problem in wet or icy conditions.
**14**	Surface Standard	P14 = 1 if acceptable P14 = 0.5 if acceptable but having issues P14 = 0 if not acceptable	Pedestrian crossing surface should be stable, firm, and slip-resistant even during rainy conditions
**15**	Dropped Curbs/Curb Ramp	P15 = if available according to standards P15 = if available but not according to standards P15= if not available	(1) Minimum ramp width at the crosswalk should be 1.2 m (2) Minimum top landing 1.2 m × 1.2 m with a slope of 2%
**16**	Tactile Paving	P16 = 1 if available according to standards P16 = 0.5 if available but not according to standards P16 = 0 if not available	It should be colored Preferable distance from the edge of the footpath, any obstruction boundary and wall = 0.60–080 m Minimum width= 0.30 m
**17**	Parking Prohibition when Curb Extension is not Provided	P17 = 1 if parking is prohibited in ≥ 15 m before crossing P17= 0.5 if parking is prohibited but not in 15 m P17= 0 if parking is not prohibited	At least 15 meters on either side of the crossing point, can be 6 meters if there are curb extensions at least 2 meters deep

**Table 4:** Description of the pedestrian crossing level of service rating and scores.

PCLOS % Rating	Score	Description
**A**	80–100	Excellent condition, most of the pedestrian crossing facilities are present
**B**	60–79	Good condition, some of the essential crossing facilities are present
**C**	40–59	Average condition, pedestrian crossing facilities are present but need attention for improvement
**D**	20–39	Bad condition, few pedestrian crossing facilities
**E**	1–19	Very bad condition, very few pedestrian crossing facilities
**F**	0	Worst condition, no pedestrian crossing facility is available

**Table 5:** Pedestrian crossing level of service ratings and scores for the study area.

Sr. No	Case Study	PCLOS Score	PCLOS%	PCLOS Grade
**1**	Ministry of Domestic Trade intersection in Putrajaya, Malaysia	52.63	82.95	A
**2**	Midblock crosswalk at the Ministry of Home Affairs, Putrajaya	48.86	77.26	B
**3**	Menara Prisma Putrajaya intersection crossing	39.26	61.82	B
**4**	Midblock crosswalk in front of the Putrajaya Corporation	37.44	59.00	C

**Table 6:** Summary of results for calculating the PCLOS.

Sr. No	Case Study	Indicator Score																	PCLOS %	PCLOS Grade
		1	2	3	4	5	6	7	8	9	10	11	12	13	14	15	16	17		
**1**	Ministry of Domestic Trade Putrajaya	1	1	1	0.5	1	1	0.5	1	1	1	1	0.5	1	1	0.5	0	1	82.95	A
**2**	Midblock crossing at the Ministry of Home Affairs Putrajaya	1	1	1	0.5	1	1	0.5	1	1	1	0.5	0	1	1	0.5	0	1	77.26	B
**3**	Menara Prisma Putrajaya intersection crossing	1	1	1	0.5	0.5	1	0	1	0	1	0	0	1	0.5	1	0	1	61.87	B
**4**	Midblock crossing in front of the Putrajaya Corporation	1	1	1	0.5	1	1	0.5	0.5	0.5	0.5	0	0	0.5	0.5	1	0	1	59	C

## Data Availability

All the essential data are shown in the manuscript.
